# Preliminary evidence for the factor structure, concurrent validity, and construct validity of the Roommate Relationship Scale in a college sample

**DOI:** 10.3389/fpsyg.2022.960421

**Published:** 2022-09-23

**Authors:** Mairéad A. Willis, Sean P. Lane

**Affiliations:** ^1^Department of Psychological Sciences, Purdue University, West Lafayette, IN, United States; ^2^Department of Psychological Sciences, University of Missouri, Columbia, MO, United States

**Keywords:** relationships, measurement, roommates, college, emerging adulthood

## Abstract

Roommate relationships are fundamental to the social environment of many emerging adults. However, no validated, widely used, measure of roommate relationship quality exists for examining the impact of these relationships on individual functioning and health. In this report, we present preliminary evidence of the factor structure, concurrent validity, and construct validity of the Roommate Relationship Scale (RRS) as a measure of roommate relationship quality using a sample of U.S. college students who participated in a multi-wave study. An exploratory factor analysis at the first wave, and confirmatory factor analyses (CFAs) with independent samples of new participants at each of two subsequent waves showed stable factor loadings and adequate fit. Moreover, the scale demonstrated good fit and reliability in a longitudinal multilevel CFA framework. The RRS significantly positively correlated with relationship length, self-esteem, extraversion, agreeableness, and conscientiousness, and negatively correlated with symptoms of anxiety and avoidant attachment style, indicating concurrent validity of the scale with respect to these constructs. Consistent with findings from other relationship types, self-reported RRS scores decrease longitudinally, both across and between semesters of academic life, indicating construct validity of the scale. We conclude that the RRS is useful for evaluating roommate relationship quality among U.S. college students, and hopefully beyond. Further research should validate the scale’s utility in other, more diverse, populations and refine its underlying generating psychological process.

## Introduction

While the impact of familial relationships and romantic partnerships on well-being has been widely studied, less attention has been paid to influential relationships in the space between late adolescence and adulthood (Erel and Burman, 1995; Hintsanen et al., 2019). In adolescence, peer relationships are thought to have a larger impact on mental health than in childhood, and there is reason to believe this influence grows in early adulthood, as many leave their families of origin and live instead with their peers (Staff et al., 2010; Bartel et al., 2020). Although emerging adults may be less likely to experience interpersonal intimacy in roommate relationships than in their families of origin, they do experience this intimacy in many cases. Importantly, roommate relationships replace familial relationships in terms of cohabitation (Erb et al., 2014). As a result, a full characterization of the social environment of emerging adults and its impacts on their well-being is incomplete without robust research on roommate relationships.

One group of emerging adults whose roommate relationships have been of particular interest to researchers are undergraduate students in the United States. According to the most current data available, approximately 16 million students are enrolled full-time in U.S. institutions of higher education (U.S. Department of Education, 2021). Many such students either choose or are randomly assigned roommates, and prior research has indicated that roommates in this population have a significant impact on one another. For example, randomly assigned college roommates have been shown to impact on one another’s otherwise embedded political beliefs (Strother et al., 2021).

In spite of researchers’ interest in the roommate relationships of college students, currently available assessments of roommate relationships are few in number and are, (1) limited in scope, (2) unvalidated in a college population, or (3) inaccessible (Bagwell et al., 2005). Prominent examples of validated scales include the Roommate Rapport Scale (Carey et al., 1988), which primarily assesses the subjective quality of roommate communication, and the Roommate Friendship Scale (Wiltz, 2003), which has not been validated in college populations. Kurtz and Sherker (2003) constructed a measure of college roommate conflict and cohesion for a study of self-other agreement that appeared to have very good internal consistency, but they did not provide the scale items or confirm its factor structure.

In order to address the need for well-validated tools, we use data from a prior academic year-long longitudinal study of initial elevation bias to provide preliminary evidence for the validity of the Roommate Relationship Scale (RRS), a measure of roommate relationship quality that has been used in prior research but has not yet been validated (West, 2008; Shrout et al., 2018; Xu and Shrout, 2018). The multi-wave study design allows us to assess scale reliability of both stable levels of and systematic change in relationship quality. It also allows us to demonstrate the relationship of the tool to constructs hypothetically related to roommate relationship quality and to evaluate if it displays a similar initial elevation bias to that observed in other self-report scales, which would be cause for concern if used in single-time-point studies (Shrout et al., 2018). Although the study did not contain comparison measures of roommate relationship quality, the unique study design does provide a scale which, contrary to the shortcomings of other extant scales, (1) is broad in the content covered by its items, (2) can be tested for preliminary evidence of concurrent and construct validity in a college population, and (3) is publicly available for use by any researcher.

Various related constructs were chosen to confirm the concurrent validity of the RRS, or its associations to measures of theoretically related constructs (Cronbach and Meehl, 1955). Prior research has indicated that romantic relationship quality is positively correlated with extraversion, agreeableness, and conscientiousness and negatively correlated with neuroticism, although these correlations are not always detected, and their magnitude varies somewhat with report method (Shaver and Brennan, 1992; Noftle and Shaver, 2006; Holland and Roisman, 2008). Although the literature on roommate relationship quality and personality is quite limited, one previous study also found a positive correlation between conscientiousness and relationship quality among college roommates (Kurtz and Sherker, 2003). Prior research on romantic couples has also shown that both anxious and avoidant attachment correlate negatively with relationship quality and with markers of relationship quality such as satisfaction (Shaver and Brennan, 1992; Noftle and Shaver, 2006). Romantic relationship quality has also been shown to correlate positively with self-esteem (Doyle and Molix, 2014) and relationship length (Erol and Orth, 2016).

Perhaps counterintuitively, the literature on associations between romantic relationship quality and symptoms of anxiety and depression is nuanced. While cross-sectional studies of the association between marital relationship quality and symptoms of depression predictably indicate the presence of a negative relationship, longitudinal studies and research on mechanisms produce mixed findings (Goldfarb and Trudel, 2019). Similarly, studies show a relationship between marital quality and anxiety, but the size of these associations increases when anxiety disorders are measured as categorical entities rather than symptom scores and has been shown to vary with the gender of the partner reporting anxiety (Leach et al., 2013; Postler et al., 2022). Finally, some research suggests that the association between relationship satisfaction and general negative affect may vary nonlinearly over time, such that the association between relationship satisfaction and negative affect depends upon the current association between relationship satisfaction and external stressors (Tesser and Beach, 1998). Given the mixed data on the relationship between marital quality and anxiety and depression symptomatology, and the less intimate nature of mostly new roommate relationships in comparison to marital relationships, it was our expectation that the RRS would not have a strong relationship to measures of depression and anxiety.

Based on the romantic relationships literature, we predicted that the RRS would demonstrate concurrent validity by correlating positively with extraversion, agreeableness, conscientiousness, relationship length, and self-esteem, negatively with neuroticism, anxious attachment style, and avoidant attachment style, and showing no relationship with openness. As previously stated, we also predicted that the RRS would show no relationship to anxious or depressive symptoms. We expected that no associations would be so large as to be redundant with relationship quality, and that in particular correlations with relationship length would be low to moderate in magnitude.

In addition to tests of concurrent validity, the longitudinal nature of the dataset provided an opportunity to examine the construct validity of the scale both by ensuring that the scale was appropriate for single-time-point use and by confirming that it behaved similarly to measures of romantic relationship quality over time (Cronbach and Meehl, 1955). Although romantic relationship quality has been shown to correlate positively with relationship length at baseline, it has also been shown to decline over time (Hirschberger et al., 2009; Lavner and Bradbury, 2010). Theoretically, this decline should also be observable in roommate relationships. Therefore, if the RRS were to decline over time, it would provide evidence of the construct validity of the scale. In addition, analyzing the trajectory of the RRS made it possible to test whether the scale demonstrates an initial elevation bias. We did not make a prediction regarding initial elevation bias of the scale, but this bias was tested for in order to determine whether any decline in relationship quality could be in part an artifact of such a bias.

## Materials and methods

### Participants

Participants were 870 college undergraduates ($M_{\mathrm{age}}\;$= 18.90, *SD* = 1.43, Range = 17–35) in a large city in the Northeastern United States recruited as part of a 4-wave panel study on initial elevation bias of self-reports (Shrout et al., 2018). As is typical for the population, roommate pairs were organized by the students and their universities rather than by the researchers. Individuals received up to $50 for their participation ($10 each for a background questionnaire and four bimonthly surveys) and had the opportunity to win one of five $250 lotteries. All procedures were approved by the University Committee on Activities Involving Human Subjects (UCAIHS) at New York University under approval number 7062, and all participants signed consent forms approved by the UCAIHS.

### Procedures

Participants completed demographic information and background measures at the beginning of the study in September 2010 (baseline). Participants then joined the focal study at one of three randomly assigned time points (October [Wave 1], December [Wave 2], or February [Wave 3]), filling out parallel measures repeated at each time point (see Shrout et al., 2018 for full description). All participants were invited to participate in the final survey in April (Wave 4). Due to the unbalanced, staggered nature of the experimental study design, the number of participants who completed the focal measures varied at each time point. Response rates were generally high such that 84.1%, 87.6%, 82.1%, and 74.0% of individuals invited to participate in the respective October, December, February, and April waves completed surveys.

### Measures

All measures selected were highly cited measures that have been utilized in prior research on relationship quality in other contexts (Noftle and Shaver, 2006; Robinson and Cameron, 2012; Givertz et al., 2013; Zheng et al., 2021; Zimmermann et al., 2021). Unless otherwise noted, individual item scores were averaged to create composite scores.

#### Baseline

##### Relationship length

Relationship length was assessed *via* two free-response items in the background questionnaire which inquired about months and years of acquaintance, respectively. These items were then combined to create a single estimate of relationship length, measured in continuous years.

##### Self-esteem

Self-esteem was assessed at baseline using the Rosenberg Self-Esteem scale (RSE; Rosenberg, 1979). The RSE is a ten-item scale with responses on a four-point scale (from 1 = *Strongly agree* to 4 = *Strongly disagree*). Items include *I feel that I’m a person of worth*, *at least on an equal plane with others* and *I feel I do not have much to be proud of*.

##### Experience with close relationships

Anxious and avoidant attachment tendencies were measured at baseline using a truncated version of the Experience with Close Relationships-Revised questionnaire comprised of seven questions from the avoidance subscale and seven from the anxiety subscale (ECR-R; Fraley et al., 2000). Participants responded on a seven-point scale (from 1 = *Strongly Disagree* to 7 = *Strongly Agree*). Items included *I prefer not to show a partner how I feel deep down* to assess avoidant attachment style and *I often worry that a partner will not want to stay with me* to assess anxious attachment style.

##### Personality

Personality was assessed at baseline using an inventory of the Big Five personality domains (John and Srivastava, 1999). Respondents endorsed items for each dimension including *Can be tense* (Neuroticism), *Is talkative* (Extraversion), *Is original*, *comes up with new ideas* (Openness), *Is helpful and unselfish with others* (Agreeableness), and *Does a thorough job* (Conscientiousness) to complete the phrase *I see myself as someone who…*. Responses were given on a five-point scale (from 1 = *Disagree Strongly* to 5 = *Agree Strongly*).

#### Repeated measures

##### Roommate relationship quality

Roommate relationship quality was measured using the RRS. The scale consists of 19 face valid items assessing roommate relationship quality on a seven or five-point Likert scale (Table 1). Items include *How much time did you spend with your roommate in the past weeks?* (from 1 = *None* to 7 = *A great deal*) and *Over the past several days, my roommate disclosed to me things about his/her personal life* (from 1 = *Not at all* to 5 = *Extremely*).

**Table 1:** Roommate relationship scale items.

Item	Item content
1	How much time did you spend with your roommate in the past weeks?
2	How confident are you of the reports you gave for your roommate?
3	My roommate really understood me over the past several days (e.g., he or she understood the type of person that I am)
4	My roommate is an excellent judge of my character (P1)
5	I am an excellent judge of my roommate’s character (P1)
6	It has been easy to express who I really am when I was with my roommate over the past few days
7^a^	I felt I had to change myself to fit in with my roommate over the past few days
8	Over the past few days, my roommate has accepted me into his/her group of friends (P2)
9	Over the past several days, I have accepted my roommate into my groups of friends (P2)
10	I want to be accepted by my roommate (P3)
11	My roommate wants to be accepted by me (P3)
12	My roommate and I are becoming close friends
13	Over the past several days, my roommate disclosed to me things about his/her personal life (P4)
14	I was completely myself when I was around my roommate over the past several days
15	Over the past several days, I disclosed to my roommate things about my personal life (P4)
16	If my roommate didn’t want to be friends with me, my feelings would be hurt
17	I want a new roommate (R)
18^a^	It would be easy for me to get a new roommate
19	My roommate and I have a lot in common

Items 1 (from 1 = *None* to 7 = *A great deal*) and 2 (from 1 = *Not very confident* to 7 = *Very confident*) were measured on a different scale than the other items (from 1 = Not at all to 5 = Extremely).

* Indicates items dropped from the final scale. (P#) indicates self-roommate reciprocal pairs of items whose residuals were a priori correlated. (R) is reverse scored.

##### Depressive symptoms

Depressive symptoms were assessed at each wave using the first eight items of the Patient Health Questionnaire-9 (PHQ-9; Kroenke et al., 2001). Respondents endorsed items including *Poor appetite or overeating* and *Trouble falling or staying asleep*, *or sleeping too much* in response to the question, *IN THE PAST 2 WEEKS*, *how often have you been bothered by any of the following problems?*. Responses were given on a four-point scale (from 1 = *Not at all* to 4 = *Nearly every day*).

##### Symptoms of anxiety

Symptoms of anxiety were measured at each wave using an 18-item version of the Zung Anxiety scale (Zung, 1971). Responses to items such as *I felt more nervous and anxious than usual* and *I felt that everything was all right and nothing bad would happen* were given on a four-point scale (from 1 = *Rarely or none of the time (<1 day)* to 4 = *Most or all of the time (5–7 days)*).

### Analytic approach

We first conducted an exploratory factor analysis (EFA) using responses from the first wave with the *psych* package in R (Revelle, 2021) to determine if a single factor structure was supported and whether there were candidate items for removal. We then used the *lavaan* package (Rosseel, 2012), to conduct a series of confirmatory factor analyses (CFA), *a priori* accounting for residual correlations between four sets of reciprocal items (Table 1). Variables were considered normal if the absolute value of their skewness was no greater than 3 and their kurtosis was no greater than 10 (Kline, 2015). In terms of model fit, CFI and TLI were considered adequate above 0.80 and good above 0.90, RMSEA was considered adequate below 0.10 and excellent below 0.05, and SRMR was considered good below 0.10 (Hu and Bentler, 1999; Kline, 2015). We conducted parallel CFAs with participants assigned to begin at Waves 1, 2 and 3, such that new participants at each wave served as independent samples to validate the factor structure of the scale. No CFA with new participants was conducted at Wave 4 because no participants were assigned to begin the study during the last wave. Finally, CFAs for each wave were repeated with both new and returning participants in order to determine whether increased power due to larger sample size would alter the underlying factor structure or fit.

After corroborating the measurement of the scale using single-level factor analyses, we conducted a multilevel factor analysis to examine scale structure at both the between-person level and within-person level over time, also using *lavaan* (Rosseel, 2012). Next, we attempted to validate the concurrent validity of the scale using external measures by conducting tests of the simple correlations between the RRS and other constructs, focusing on person-level aggregates across all waves, using the *stats* package in R (R Core Team, 2022). We then tested the construct validity of the scale by analyzing longitudinal trajectories of the RRS as a function of time by conducting a series of multilevel analyses using the *lme4* and *lmerTest* packages in R (Bates et al., 2015; Kuznetsova et al., 2017). First, we conducted an analysis in which Wave, as a categorical indicator, predicted roommate relationship reports, with random intercepts for participant and roommate dyad. In order to rule out the possibility that any decline in the RRS was the result of confounding variables associated with the academic calendar, we explored whether two other aspects of timing, semester (fall or spring) and time within semester (early or late), were associated with systematic differences in roommate relationship ratings. These analyses aimed to investigate whether seasonal changes or changes in student experience over the course of a semester affected students’ perception of their relationship with their roommate. In all multilevel analyses we also accounted for possible initial elevation bias (Shrout et al., 2018).

## Results

### Participant characteristics

Approximately 75% of participants identified as female and 25% as male. Participants identified their ethnicity as Hispanic or Latino (*n* = 88, 10.4%) or Not Hispanic or Latino (*n =* 757, 89.6%) and their race as American Indian or Alaska Native (*n* = 10, 1.2%), Asian (*n* = 241, 28.4%), Black or African American (*n* = 52, 6.1%), Native Hawaiian or other Pacific Islander (*n* = 6, 0.71%), White (*n =* 526, 61.9%), or another racial identity (*n* = 101, 11.9%).^1^ The majority of students joined with a roommate (*n* = 742, 85.3%), while those remaining joined without a roommate (*n* = 128, 14.7%). Sample characteristics are described in Table 2.^2^

**Table 2:** Participant characteristics.

Age	*M* = 18.90 (*SD* = 1.43, Range = 17–35)
*Gender*	
Men	24.9%
Women	75.1%
*Ethnicity*	
Hispanic or Latino	10.4% (*n* = 88)
Not Hispanic or Latino	89.6% (*n* = 757)
*Racial identity* ^1^	
American Indian or Alaska Native	1.2% (*n* = 10)
Asian	28.4% (*n* = 241)
Black or African American	6.1% (*n* = 52)
Native Hawaiian or other Pacific Islander	0.71% (*n* = 6)
White	61.9% (*n* = 526)
Other	11.9% (*n* = 101)
*Roommate status*	
Joined with roommate	85.3% (*n* = 742)
Joined without roommate	14.7% (*n* = 128)

### Measure characteristics

Internal consistency of the RRS was high in this sample (Tables 3, 4). All background measures showed adequate to good internal consistency, and all repeated measures showed good to high internal consistency (Supplementary Tables S2, S3). Multivariate normality of all scales was evaluated by calculating skewness and kurtosis. Relationship length was found to be non-normal and was transformed prior to tests of concurrent validity (Supplementary Table S4; Table 5).

**Table 3:** Factor loadings and fit indices by Wave for groups of new and all participants.

Item	New			All		
	Wave 1	Wave 2	Wave 3	Wave 2	Wave 3	Wave 4
1	1.32	1.18	1.54	1.32	1.46	1.67
2	0.84	0.89	0.91	0.92	0.88	1.00
3	0.90	0.91	1.06	0.96	1.07	1.11
4	0.93	0.96	1.10	1.00	1.04	1.15
5	0.63	0.69	0.72	0.72	0.70	0.83
6	1.03	0.98	1.11	1.02	1.09	1.24
8	1.15	0.96	1.08	1.04	1.10	1.20
9	1.08	0.93	1.08	1.00	1.08	1.19
10	0.83	0.76	0.98	0.82	0.97	1.06
11	0.80	0.66	0.88	0.75	0.82	0.97
12	1.15	1.11	1.33	1.16	1.26	1.39
13	0.91	0.93	1.02	1.02	1.03	1.20
14	0.95	0.76	1.00	0.83	0.89	0.99
15	0.99	0.92	1.17	1.05	1.15	1.19
16	0.85	0.94	1.21	0.93	1.12	1.18
17r	0.47	0.45	0.52	0.46	0.51	0.58
19	1.02	0.94	1.01	0.98	1.03	1.06
*Fit*						
*N*	213	241	203	439	573	519
CFI	0.86	0.88	0.89	0.89	0.91	0.92
TLI	0.83	0.85	0.88	0.87	0.89	0.90
RMSEA	0.13	0.11	0.12	0.11	0.11	0.11
SRMR	0.06	0.06	0.05	0.05	0.05	0.04
ω	0.95	0.94	0.96	0.95	0.96	0.96

All loadings were statistically significant at *p* < 0.001.

**Table 4:** Multilevel factor loadings and reliabilities.

Item	Within	Between
1	0.63	1.42
2	0.38	0.84
3	0.78	0.75
4	0.64	0.83
5	0.40	0.62
6	0.82	0.80
8	0.53	1.04
9	0.53	1.01
10	0.51	0.83
11	0.39	0.79
12	0.59	1.17
13	0.54	0.97
14	0.67	0.65
15	0.65	0.93
16	0.44	1.02
17r	0.37	0.39
19	0.47	0.93
ω	0.92	0.98

All loadings were significant at *p* < 0.001. Model fit indices included, CFI = 0.91, TLI = 0.89, RMSEA = 0.07, ${\mathrm{SRMR}}_{\mathrm{Within}}$ = 0.08, ${\mathrm{SRMR}}_{\mathrm{Between}}$ = 0.06. *N*_Observations_ *=* 1,744; *N*_Participants_ *=* 700.

**Table 5:** Validity construct correlation matrix.

		*M*	*SD*	*df_1_*	1	2	3	4	5	6	7	8	9	10	11
1.	RRS	3.65	1.06		1.00										
2.	Relationship Length	0.88	0.85	1410	0.30***	1.00									
3.	Rosenberg Esteem	3.11	0.50	1828	0.10***	−0.01	1.00								
4.	PHQ-9	1.67	0.56	1831	−0.04†	0.01	−0.43***	1.00							
5.	Zung Anxiety	1.58	0.39	1825	−0.06**	0.03	−0.40***	0.74***	1.00						
6.	ECR Anxiety	3.93	1.24	1820	0.00	0.00	−0.36***	0.22***	0.22***	1.00					
7.	ECR Avoidance	3.52	1.31	1820	−0.07**	−0.04†	−0.28***	0.23***	0.19***	0.24***	1.00				
8.	Extraversion	3.38	0.83	1828	0.10***	−0.04†	0.39***	−0.14***	−0.07***	−0.16***	−0.23***	1.00			
9.	Agreeableness	3.71	0.64	1828	0.18***	0.05*	0.24***	−0.17***	−0.18***	−0.17***	−0.29***	0.14***	1.00		
10.	Conscientiousness	3.53	0.67	1825	0.10***	0.10***	0.41***	−0.27***	−0.23***	−0.20***	−0.19***	0.15***	0.25***	1.00	
11.	Neuroticism	3.02	0.74	1828	−0.02	0.05**	−0.55***	0.37***	0.43***	0.39***	0.15***	−0.21***	−0.28***	−0.17***	1.00
12.	Openness	3.85	0.61	1825	0.01	−0.07***	0.22***	−0.04†	−0.02	−0.04*	−0.10***	0.26***	0.12***	0.10***	−0.12***

*M*, mean; *SD*, standard deviation; *df_1_*, degrees of freedom for the test of the correlation between the measure in question and the RRS; RRS, Roommate Relationship Scale; PHQ-9, Patient Health Questionnaire-9 scale; ECR, Experiences in Close Relationships-Revised Scale. Zung Anxiety & PHQ9 were assessed at each wave; all other scales were filled out as background measures. Relationship length underwent a square-root transformation to achieve normality.

*** *p* < 0.001;

** *p* < 0.01;

* p < 0.05;

† p < 0.1.

### Exploratory factor analysis of Wave 1

**Figure 1:**
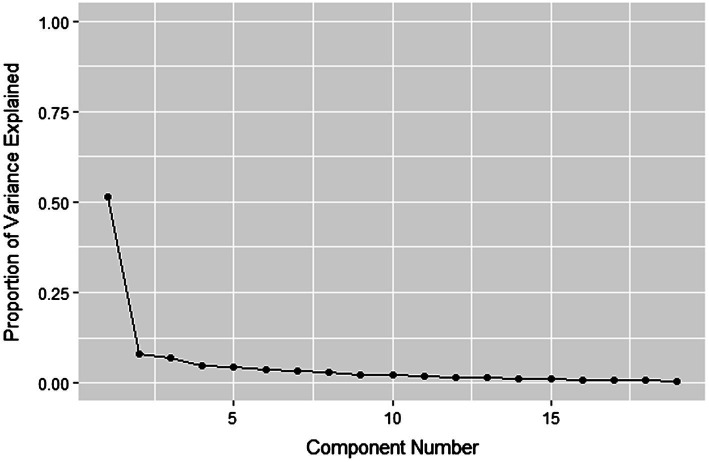
Exploratory factor analysis of the RRS with Wave 1 participants. This scree-plot provides a visual representation of the compelling evidence for a single-factor solution found in the EFA with Wave 1 participants.

Exploratory factor analysis was conducted using a principle components approach to extract five factors (Bartlett’s sphericity test = 3006.55, *df* = 171, *p* < 0.001; KMO = 0.92). Results indicated clear evidence of a single underlying factor (1^st^ eigenvalue = 9.37, 49% variance; >1^st^ eigenvalues <1.12, variances <7%) (Figure 1; Table 6). Additionally, the latent factor did not load onto two face-ambiguous items (7, 18; Tables 1, 6), which were dropped from subsequent CFAs. The analysis further indicated that one item (17) loaded negatively onto the latent factor. This item was reverse-coded in the subsequent CFAs (Tables 1, 6).

**Table 6:** Exploratory factor analysis of the 19-item RRS.

Item	Factor loading
1. How much time did you spend with your roommate in the past weeks?	0.73
2. How confident are you of the reports you gave for your roommate?	0.61
3. My roommate really understood me over the past several days (e.g., he or she understood the type of person that I am)	0.82
4. My roommate is an excellent judge of my character	0.81
5. I am an excellent judge of my roommate’s character	0.68
6. It has been easy to express who I really am when I was with my roommate over the past few days	0.79
7. I felt I had to change myself to fit in with my roommate over the past few days	−0.25
8. Over the past few days, my roommate has accepted me into his/her group of friends	0.81
9. Over the past several days, I have accepted my roommate into my groups of friends	0.79
10. I want to be accepted by my roommate	0.70
11. My roommate wants to be accepted by me	0.72
12. My roommate and I are becoming close friends	0.88
13. Over the past several days, my roommate disclosed to me things about his/her personal life	0.70
14. I was completely myself when I was around my roommate over the past several days	0.75
15. Over the past several days, I disclosed to my roommate things about my personal life	0.73
16. If my roommate did not want to be friends with me, my feelings would be hurt	0.62
17. I want a new roommate	−0.51
18. It would be easy for me to get a new roommate	−0.02
19. My roommate and I have a lot in common	0.81
*Factor indices*	
Eigenvalues	9.37
ω	0.94
Means (SD)	3.44 (0.81)
Total explained variance (%)	49%

Extraction method, principle components; Rotation method, none.

### Single-level confirmatory factor analyses

Factor structure and model fit was consistent across waves, with marginal to moderately good fit indicated by the Comparative Fit Index (CFI), Tucker-Lewis Index (TLI), root mean square error of approximation (RMSEA), and standardized root mean square residual (SRMR) (Table 3). Increases in sample size, in terms of including individuals who participated in multiple waves, did not substantively affect factor structure but was necessarily associated with marginally better fit, indicating general stability of the scale structure.

### Multilevel confirmatory factor analysis

Multilevel confirmatory factor analysis indicated that loadings were notably higher at the between-person than the within-person level. However, all within-person loadings were above 0.30 and statistically significant (*p* < 0.001), indicating independent consistent structure. Model fit was good, indicating that accounting for within-person variability improved the fit of the factor analysis. A majority of variance was accounted for at the between-person level (72.88%), with less explained at the within-person level (34.02%). Estimated omega reliabilities were excellent for between-person (*ω* = 0.98) and within-person scale scores (*ω* = 0.92) suggesting that the RRS can be used to reliably detect both trait-like differences and longitudinal changes in roommate relationship quality (Table 4).

### Concurrent validity of the RRS with respect to measures of related constructs

Concurrent validity was assessed using simple correlation tests. The correlations between the RRS and relationship length, Rosenberg self-esteem, Zung anxiety, avoidant attachment, extraversion, agreeableness, and conscientiousness were all statistically significant in the expected directions. Counter to our predictions, no significant relationship was detected between the RRS and anxious attachment style or neuroticism, and a significant correlation was detected between the RRS and symptoms of anxiety. As expected, there was no significant relationship between the RRS and either openness or symptoms of depression (Table 5).

### Construct validity of the RRS with respect to its behavior over time

**Figure 2:**
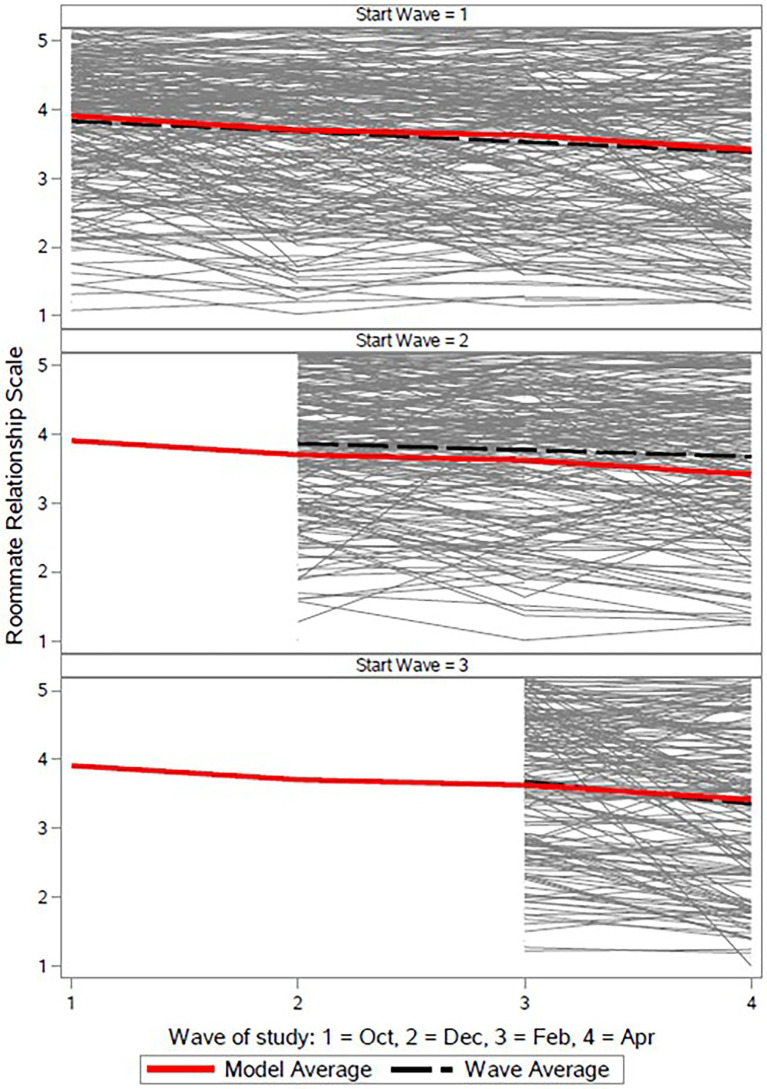
Individual and model estimated average trajectories of the Roommate Relationship Scale across the starting waves. Individual trajectories in each panel reflect participants who started at the first (Top panel), second (Middle panel), and third waves (Bottom panel), respectively. The red line in each panel indicates the multilevel linear model estimate across all participants and waves, while the black line in each panel represents the multilevel linear model estimate for participants who began the study at the wave specified in the panel title.

Multilevel model results indicated no significant effect of participants’ first response on RRS scores (*b* = 0.07, SE = 0.04, *p* = 0.073). This indicates that the significant negative relationship between the RRS and each wave after Wave 1 reflects a true depreciation in relationship quality over the course of the academic year rather than an initial elevation bias. In comparison to the first wave, roommate relationship quality was lower at each subsequent wave. The estimated sample mean reported RRS at Wave 1 was 3.82 (SE = 0.08, *p* < 0.001), which steadily decreased at Waves 2 (*b* = −0.16, SE = 0.05, *p* = 0.001), 3 (*b* = −0.22, SE = 0.06, *p* < 0.001), and 4 (*b* = −0.40, SE = 0.07, *p* < 0.001; Figure 2). Random intercept effects indicated that there was both substantial between individual (*σ*^2^ = 0.17, *Χ*^2^(1) = 159.44, *p* < 0.001, ICC = 0.15) and roommate-pair (*σ*^2^ = 0.73, *Χ*^2^(1) = 221.18, *p* < 0.001, ICC = 0.64) variability in RRS average levels across the academic year, with the latter indicating high agreement in roommates’ ratings with one another.

In addition, we fit an alternative nested model with two binary indicators signifying Fall/Spring semester and early/late within each semester. The estimated sample mean early in the fall semester was 3.83 (SE = 0.07, *p* < 0.001), and it decreased independently as a function of later in the semester waves (e.g., December/April; *b* = −0.18, SE = 0.03, *p* < 0.001), and in the Spring semester (*b* = −0.24, SE = 0.04, *p* < 0.001). Finally, we estimated a further nested model that treated wave as a linear effect. The Wave 1 intercept was 3.81 (SE = 0.07, *p* < 0.001), and there was a negative slope spanning the academic year (*b* = −0.13, SE = 0.02, *p* < 0.001).^3^ Importantly, likelihood ratio tests indicated that while the semester/seasonal model fit better than the linear model [*Χ*^2^(1) = 5.64, *p* = 0.018], the categorical model did not improve on the fit of the semester/seasonal model [*Χ*^2^(1) = 0.22, *p* = 0.641].

## Discussion

The results of our factor analyses support that the RRS measures a unidimensional construct. In addition, it shows substantial dyad-level consistency over time, meaning that roommates demonstrate stable agreement on the subjective quality of their relationship with one another. As expected, scores on the RRS appear to decrease over time, indicating the construct validity of the scale. Decrease in marital satisfaction after marriage, on average, is a finding that has been replicated, and this decrease has been demonstrated to continue over years and even decades (Hirschberger et al., 2009; Lavner and Bradbury, 2010). The findings of this study indicate that college roommates follow a similar trajectory, reporting that they perceive decreased quality in their roommate relationship over time, even when a bias toward higher initial reports is accounted for. In addition, the academic year contains unique characteristics, such as overlapping with a seasonal change which has been shown to impact mental health and containing stressful exam periods at the end of each semester (Madden et al., 1996; Wehr, 1998; Thompson and Bolger, 1999). While a multilevel model accounting for these seasonal and semester changes fit better than a model that treated time as a continuous variable, its fit did not differ significantly from a model that treated time as a categorical variable, with four values representing each of the study waves. This indicates that while treating time as a continuous variable missed important nuance inherent in the different times of the academic year when study waves occurred, differentiating further to capture theoretical stressors such as seasonal changes and exam periods was unnecessary in this sample.

Also as predicted, the scale was significantly positively correlated with extraversion, agreeableness, conscientiousness, relationship length, and self-esteem, negatively correlated with avoidant attachment style, and showed no relationship to depressive symptoms. These results provide evidence for the concurrent validity of the scale, as they demonstrate marginal, but not overlapping, correlations, given attenuation for unreliability, with constructs that have been shown to be related to relationship quality in other contexts. It also demonstrated a lack of correlation with constructs that have been shown to be generally unrelated to relationship quality previously. Importantly, no correlations were so large in magnitude as to suggest redundancy between the RRS and any other constructs measured, particularly given reliability disattenuation.

Counter to our predictions, we did not detect a significant relationship with neuroticism or anxious attachment style, and we did detect a negative relationship with symptoms of anxiety. The small and non-significant relationships between avoidant and anxious attachment, respectively, and the RRS also appear to deviate from findings in the literature on romantic relationships, where associations between attachment style and relationship quality are higher than associations between personality and relationship quality (Shaver and Brennan, 1992; Noftle and Shaver, 2006). This may indicate that attachment styles, having been developed in large part in the literature on romantic relationships, are not as relevant to largely non-romantic roommate relationships, or that attachment style differs based on the contextual relationship (Asendorpf et al., 1997; Welch and Houser, 2010). The small, significant association detected between relationship quality and symptoms of anxiety may indicate that given the high statistical power of this study, it was possible to detect a relationship that much prior research has not been powered to detect. Whether such an effect would be clinically meaningful, however, is a question for further study.

Finally, and counter to our prediction, no significant correlation between the RRS and neuroticism was present in our sample. In general, research has indicated that neuroticism is a relatively robust indicator of romantic relationship outcomes such as relationship quality and satisfaction (Holland and Roisman, 2008). However, some research indicates that particular subdomains of neuroticism may be significantly more associated with relationship quality than others, such that associations between neuroticism and relationship quality depend not only on the average neuroticism in a sample but also on the composition of that trait (Noftle and Shaver, 2006). In addition, perhaps because personality traits have more overlap with undifferentiated than relationship-specific affect, personality traits in general have been observed to have weaker associations with relationship outcomes than attachment styles (Shaver and Brennan, 1992). Measurement method and relationship type may also affect the magnitude of the observed relationship between neuroticism and relationship quality, with one study finding a significant relationship when neuroticism was reported by the partner, but not when it was self-reported among dating couples. The pattern was reversed among married couples, while the effects were of similar magnitude among engaged couples (Holland and Roisman, 2008). Given that research on roommate relationships is relatively limited, it remains for future studies to confirm whether neuroticism is consistently unlinked to roommate relationship quality across a variety of methods and samples, and whether this is a point of difference between roommate and romantic relationships.

### Future directions

This preliminary evidence of the factor structure, concurrent validity, and construct validity of the RRS in a college setting opens the door for a number of future investigations. First, due to the single participant pool used to validate the RRS in this study, further research examining psychometric properties of the scale in different populations is required in order for the scale to be robustly validated. In addition, the exact nature of the construct measured by the RRS remains unclear. The content of the items includes diverse constructs including disclosure, self-confidence in judgments, and time spent with one’s roommate in the recent past. We have referred to the RRS as a measure of perceived relationship quality, which appears to best capture the content of the scale. However, future research could determine the nature of the construct more precisely by comparing the RRS, and in particular its convergence with related scales, to that of other measures of friendship and roommate relationship. Finally, the scale may be of use in further investigating convergence between roommates in their relationship ratings.

### Limitations

Because this analysis was carried out with extant data, it was not possible to compare the validity of the RRS to that of the Roommate Rapport Scale, the Roommate Friendship Scale, or the Roommate Relationship Questionnaire (Carey et al., 1988; Kurtz and Sherker, 2003; Wiltz, 2003). We expect that these scales will correlate with one another, and future research should confirm that these scales assess separable constructs. In addition, the single location and somewhat homogenous sample may limit the generalizability of this scale validation. For example, the high cost and limited availability of housing in an urban setting may have required participants in this sample to join or remain in roommate relationships at a higher rate than would be typical for people in a smaller city or town. In addition, while differences between same-race and interracial roommate relationships have been explored in the literature, to our knowledge there is no literature to draw from to predict how the construct of roommate relationship quality might differ in groups with different composition in terms of racial identity (Shook and Fazio, 2008; Bresnahan et al., 2009; Trail et al., 2009). The literature also indicates that roommate relationships and their correlation to constructs including depression differ based on gender, meaning that the structure of roommate relationship quality might differ in samples with different gender composition (Siegel and Alloy, 1990; Joiner and Metalsky, 1995; Ansell et al., 2008). Finally, although extensive studies on the subject are not available, theoretically it is plausible that as payment motivates participation in studies, it may also alter the characteristics of the participant pool (Nelson and Merz, 2002). Socioeconomic characteristics of the sample are available in the supplement (Supplementary Tables S5–S7). Given the novel nature of the RRS and the single study used to investigate here, it is our belief that the best way to fully explore the effect of differing demographics on the structure of the RRS is to utilize it with new samples.

## Conclusion

Our results indicate that the RRS shows promise as a measure of roommate relationship quality in the context of college student relationships. First, an exploratory factor analysis with new participants at Wave 1 of our study indicated that a single-factor solution is most appropriate for the scale, and factor loadings indicated that two items in the scale should be dropped and one reverse-coded. Then, CFAs using new participants at Waves 2 and 3 indicated that the single-factor solution suggested by the EFA had adequate to marginal fit in two new samples of participants. Finally, a multilevel CFA which took advantage of the repeated measurement of participants in the dataset demonstrated good model fit. Once the factor structure of the RRS in this sample had been confirmed, correlation analyses were performed in order to assess the concurrent validity of the RRS with respect to measures of related constructs. These tests indicated that the RRS was related as expected to the majority of constructs proposed, and that none of these relationships were so large as to indicate that the RRS was redundant with any of these measures. Finally, evidence for the construct validity of the RRS was provided by multilevel analyses of changes in the RRS over time. RRS score at each time point predicted a decrease in RRS score at the subsequent timepoint, as observed in the romantic relationships literature. This change was not due to an initial elevation bias in this sample. However, in order for the RRS can be considered a robustly validated tool of roommate relationship quality across contexts, further studies must demonstrate its psychometric properties in samples which differ widely from the sample used here and from one another in terms of demographic characteristics and life contexts.

## Data availability statement

The data analyzed in this study is subject to the following licenses/restrictions: The data used in this study are restricted and can be accessed only with permission from the authors. Requests to access these datasets should be directed to SL, lanesp@missouri.edu.

## Ethics statement

The studies involving human participants were reviewed and approved by University Committee on Activities Involving Human Subjects (UCAIHS) at New York University. Written informed consent to participate in this study was provided by the participants or their legal guardian/next of kin.

## Author contributions

MW conducted initial analyses and wrote the initial draft of the manuscript. MW and SL contributed to the study conception, manuscript revision, and submission. All authors contributed to the article and approved the submitted version.

## Funding

This research was supported by the National Institutes of Health research grants R01 AA017672 (Shrout) and R01 AA027264 (Lane/Hennes).

## Conflict of interest

The authors declare that the research was conducted in the absence of any commercial or financial relationships that could be construed as a potential conflict of interest.

## Publisher’s note

All claims expressed in this article are solely those of the authors and do not necessarily represent those of their affiliated organizations, or those of the publisher, the editors and the reviewers. Any product that may be evaluated in this article, or claim that may be made by its manufacturer, is not guaranteed or endorsed by the publisher.

## Footnotes

^1^Participants were able to identify with one or more racial identities or to decline to respond regarding any demographic characteristic. Percentages given are the percentage of participants responding to a question who identified with the identity in question.

^2^Chi-squared tests indicated that the samples for individual waves did not differ from one another on any demographic characteristics. See Supplementary Table S1 for more details.

^3^Random intercepts and corresponding ICCs for individuals (σ^2^ = 0.17, *ICC = 0*.15) and roommate dyads (σ^2^ = 0.73, *ICC =* 0.64) remained essentially unchanged for the two alternative models examining trajectories by wave.
