# Supplementary material for: Preliminary evidence for the factor structure, concurrent validity, and construct validity of the Roommate Relationship Scale in a college sample
Source: Front Psychol. 2022 Sep 23;13:960421. doi: 10.3389/fpsyg.2022.960421 (PMC9537060; doi:10.3389/fpsyg.2022.960421)
Supplement: Supplementary file 1 [file Table_1.docx]

Supplementary Material

# Supplementary Tables

| **Table S1.** Chi-squared tests of differences in demographic characteristics between waves | | | |
| --- | --- | --- | --- |
| *Characteristic* | *χ^2^* | *df* | p*-value* |
| Age | 12.30 | 33 | >.999 |
| Gender | 0.43 | 3 | .933 |
| Ethnicity | 0.17 | 3 | .982 |
| *Racial Identity* | | | |
| American Indian or Alaska Native | 0.05 | 3 | .997 |
| Asian | 0.07 | 3 | .996 |
| Black or African American | 1.22 | 3 | .749 |
| Native Hawaiian or other Pacific Islander | 0.31 | 3 | .959 |
| White | 0.15 | 3 | .985 |
| Other racial identity | 0.58 | 3 | .900 |

| **Table S2 Reliability of background measures.** | |
| --- | --- |
| *Scale* | ω |
| Rosenberg Self-Esteem | .89 |
| Anxious Attachment Style | .85 |
| Avoidant Attachment Style | .88 |
| Extraversion | .89 |
| Agreeableness | .80 |
| Conscientiousness | .83 |
| Neuroticism | .82 |
| Openness | .83 |

| **Table S3 Reliability of repeated measures.** | | | | | |
| --- | --- | --- | --- | --- | --- |
| *Scale* | *Wave 1* | *Wave 2* | *Wave 3* | *Wave 4* | *Overall* |
|  | ω | ω | ω | ω | ω |
| Patient Health Questionnaire-9 | .84 | .88 | .87 | .88 | .87 |
| Zung Anxiety | .87 | .86 | .85 | .84 | .85 |

| **Table S4. Skewness and kurtosis of study scales.** | | |
| --- | --- | --- |
| *Item* | *Skewness* | *Kurtosis* |
| *RRS* | -0.49 | -0.70 |
| *Relationship Length* | 4.13 | 20.31 |
| *Square root transformed relationship length* | 2.01 | 4.50 |
| *Rosenberg Esteem* | -0.22 | -0.35 |
| *PHQ-9* | 1.30 | 1.90 |
| *Zung Anxiety* | 1.24 | 1.97 |
| *ECR Anxiety* | -0.08 | -0.48 |
| *ECR Avoidance* | 0.26 | -0.43 |
| *Extraversion* | -0.14 | -0.61 |
| *Agreeableness* | -0.56 | 0.60 |
| *Conscientiousness* | -0.17 | -0.09 |
| *Neuroticism* | 0.04 | -0.41 |
| *Openness* | -0.53 | 0.19 |

| **Table S5.** Self-reported Family Socioeconomic Status (SES)^^[[1]](#footnote-1)^^ | |
| --- | --- |
| *SES* | *Frequency* |
| Working class | 73 |
| Middle class | 316 |
| Upper middle class | 390 |
| Upper class | 37 |
| Don’t know/prefer not to say | 34 |

| **Table S6.** Parents’ highest education^^[[2]](#footnote-2)^^ | |
| --- | --- |
| *Education level* | *Frequency* |
| Less than high school | 18 |
| High school diploma | 157 |
| Some college (including Associates Degree) | 113 |
| B.A./B.S. | 391 |
| M.A./M.S. | 239 |
| M.D. | 89 |
| Ph.D. | 110 |
| MBA | 54 |
| J.D. | 35 |
| Other | 14 |

| **Table S7.** Chi-squared tests of differences in socioeconomic status between waves | | | |
| --- | --- | --- | --- |
|  | *χ^2^* | *df* | p*-value* |
| Family SES | 1.94 | 12 | >.999 |
| *Parents’ highest education* | | | |
|  | *χ^2^* | *df* | p*-value* |
| Less than high school | 0.40 | 3 | .940 |
| High school diploma | 1.06 | 3 | .787 |
| Some college (including Associates Degree) | 0.08 | 3 | .994 |
| B.A./B.S. | 0.50 | 3 | .918 |
| M.A./M.S. | 3.07 | 3 | .381 |
| M.D. | 0.59 | 3 | .899 |
| Ph.D. | 0.97 | 3 | .808 |
| MBA | 1.28 | 3 | .733 |
| J.D. | 0.09 | 3 | .993 |
| Other | 1.15 | 3 | .764 |

# Supplementary Code

#Number of participants who joined w/ a roommate vs alone

dyadfreqs<-table(CLS$dyadnum)

dyadfreqs<-as.data.frame(dyadfreqs)

table(dyadfreqs$Freq)

128 #No. of participants without a roommate

128/(128+742)

371*2 #No. of participants with a roommate

742/(128+742)

#Separating out data by wave

CLS1 <- CLS[which(CLS$WAVE == 1), ]

CLS2 <- CLS[which(CLS$WAVE == 2), ]

CLS3 <- CLS[which(CLS$WAVE == 3), ]

CLS4 <- CLS[which(CLS$WAVE == 4), ]

#Determining response rate by wave

#Participant start assignments

table(CLS$WAVE,CLS$XFIRST)

# 0 1

# 1 0 295

# 2 295 285

# 3 580 290

# 4 870 0

#Participants consenting per wave

table(CLS$WAVE,CLS$consent)

# 1 2

#1 248 0

#2 508 1

#3 714 1

#4 644 0

#Wave 1 response rate

248/295

#Wave 2 response rate

508/(295+285)

#Wave 3 response rate

714/(295+285+290)

#Wave 4 response rate

644/(295+285+290)

#Demographic data

Demo1 <- CLS1[which(CLS1$consent == 1),]

Demo2 <- CLS2[which(CLS2$consent == 1),]

Demo3 <- CLS3[which(CLS3$consent == 1),]

Demo4 <- CLS4[which(CLS4$consent == 1),]

#Overall age breakdown (Using both those who participated and those who did not participate at Wave 1 (ie, all participants))

table(CLS1$bage)

mean(na.omit(CLS1$bage))

sd(na.omit(CLS1$bage))

#Overall gender

table(CLS$WAVE,CLS$bgender)

211/(211+637)

#Overall ethnicity

table(CLS$WAVE,CLS$bhispanic)

88/(88+757)

#Overall racial identity

table(CLS$WAVE,CLS$beth01) #American Indian or Alaska Native

10/(840+10)

table(CLS$WAVE,CLS$beth02) #Asian

241/(609+241)

table(CLS$WAVE,CLS$beth03) #Black or African American

52/(798+52)

table(CLS$WAVE,CLS$beth04) #Native Hawaiian or Other Pacific Islander

6/(844+6)

table(CLS$WAVE,CLS$beth05) #white

526/(324+526)

table(CLS$WAVE,CLS$beth06) #Other

101/(749+101)

#Checking demographic imbalance between waves

#Age

Demo <- CLS[which(CLS$consent == 1),]

table(Demo$WAVE, Demo$bage)

chisq.test(Demo$WAVE, Demo$bage, correct=FALSE)

#Gender

table(Demo$WAVE,Demo$bgender)

chisq.test(Demo$WAVE, Demo$bgender, correct=FALSE)

#Ethnicity

table(Demo$WAVE,Demo$bhispanic)

chisq.test(Demo$WAVE,Demo$bhispanic,correct=FALSE)

#Racial identity

table(Demo$WAVE,Demo$beth01)

chisq.test(Demo$WAVE,Demo$beth01,correct=FALSE)

table(Demo$WAVE,Demo$beth02)

chisq.test(Demo$WAVE,Demo$beth02,correct=FALSE)

table(Demo$WAVE,Demo$beth03)

chisq.test(Demo$WAVE,Demo$beth03,correct=FALSE)

table(Demo$WAVE,Demo$beth04)

chisq.test(Demo$WAVE,Demo$beth04,correct=FALSE)

table(Demo$WAVE,Demo$beth05)

chisq.test(Demo$WAVE,Demo$beth05,correct=FALSE)

table(Demo$WAVE,Demo$beth06)

chisq.test(Demo$WAVE,Demo$beth06,correct=FALSE)

#SES

table(Demo$WAVE,Demo$bSES)

chisq.test(Demo$WAVE,Demo$bSES,correct=FALSE)

table(CLS1$bSES) #Calculating SES for the sample at the first wave gives us the overall demographics of the sample, as background characteristics were stored for each participant at each wave, regardless of whether they participated

#Parents' education

table(Demo$WAVE,Demo$beduc01)

chisq.test(Demo$WAVE,Demo$beduc01,correct=FALSE)

table(Demo$WAVE,Demo$beduc02)

chisq.test(Demo$WAVE,Demo$beduc02,correct=FALSE)

table(Demo$WAVE,Demo$beduc03)

chisq.test(Demo$WAVE,Demo$beduc03,correct=FALSE)

table(Demo$WAVE,Demo$beduc04)

chisq.test(Demo$WAVE,Demo$beduc04,correct=FALSE)

table(Demo$WAVE,Demo$beduc05)

chisq.test(Demo$WAVE,Demo$beduc05,correct=FALSE)

table(Demo$WAVE,Demo$beduc06)

chisq.test(Demo$WAVE,Demo$beduc06,correct=FALSE)

table(Demo$WAVE,Demo$beduc07)

chisq.test(Demo$WAVE,Demo$beduc07,correct=FALSE)

table(Demo$WAVE,Demo$beduc08)

chisq.test(Demo$WAVE,Demo$beduc08,correct=FALSE)

table(Demo$WAVE,Demo$beduc09)

chisq.test(Demo$WAVE,Demo$beduc09,correct=FALSE)

table(Demo$WAVE,Demo$beduc10)

chisq.test(Demo$WAVE,Demo$beduc10,correct=FALSE)

#Overall parents' education; participants were allowed to select more than one degree for the parent who had the most education

table(CLS1$beduc01)

18/(832+18)

table(CLS1$beduc02)

157/(693+157)

table(CLS1$beduc03)

113/(737+113)

table(CLS1$beduc04)

391/(459+391)

table(CLS1$beduc05)

239/(611+239)

table(CLS1$beduc06)

89/(761+89)

table(CLS1$beduc07)

110/(740+110)

table(CLS1$beduc08)

54/(796+54)

table(CLS1$beduc09)

35/(815+35)

table(CLS1$beduc10)

14/(836+14)

#Recoding months and years known roommate into a single variable

oldvalues4 <- c("","0","1.5","10","11","12","13","14","18","19","2","20","22 years","23","3","4","5","6","7","8","o","one")

newvalues4 <- c("0","0","1.5","10","11","12","13","14","18","19","2","20","22","23","3","4","5","6","7","8","NA","1")

oldvalues5 <- c("","<2","0","1","1 1/2","1 and a ha","1.5","1.7","1.8","10","11","14","16","2","2 1/2 mont","2.5","3","3 months","4","5","5 months","6","7","8","9","about 2 mo","four","less than","two")

newvalues5 <- c("0","NA","0","1","1.5","1.5","1.5","1.7","1.8","10","11","14","16","2","2.5","2.5","3","3","4","5","5","6","7","8","9","2","4","NA","2")

CLS$bLS04n <- newvalues4[match(CLS$bLS04, oldvalues4)]

CLS$bLS05n <- newvalues5[match(CLS$bLS05, oldvalues5)]

CLS$bLS04n<- as.numeric(CLS$bLS04n)

CLS$bLS05n<- as.numeric(CLS$bLS05n)

CLS$Timeknwn <- CLS$bLS04n + CLS$bLS05n/12

mean(na.omit(CLS$Timeknwn))

sd(na.omit(CLS$Timeknwn))

#Calculating internal consistency

#Background

which( colnames(CLS)=="bRSEr01")

which( colnames(CLS)=="bRSEr02")

which( colnames(CLS)=="bRSE03")

which( colnames(CLS)=="bRSEr04")

which( colnames(CLS)=="bRSE05")

which( colnames(CLS)=="bRSEr06")

which( colnames(CLS)=="bRSEr07")

which( colnames(CLS)=="bRSE08")

which( colnames(CLS)=="bRSE09")

which( colnames(CLS)=="bRSE10")

CLS_bRSE<- CLS[,c(18,20,23:25,1091:1095)]

psych::omega(CLS_bRSE,nfactors=1,seed=1)

which( colnames(CLS)=="bECR02")

which( colnames(CLS)=="bECR05")

which( colnames(CLS)=="bECR06")

which( colnames(CLS)=="bECRr08")

which( colnames(CLS)=="bECR10")

which( colnames(CLS)=="bECR12")

which( colnames(CLS)=="bECR14")

CLS_bECRanx<- CLS[,c(214,217:218,1177,222,224,226)]

psych::omega(CLS_bECRanx, nfactors=1, seed=1)

which( colnames(CLS)=="bECRr01")

which( colnames(CLS)=="bECR03")

which( colnames(CLS)=="bECR04")

which( colnames(CLS)=="bECR07")

which( colnames(CLS)=="bECRr09")

which( colnames(CLS)=="bECR11")

which( colnames(CLS)=="bECRr13")

CLS_bECRav<- CLS[,c(215:216,219,1174:1176,223)]

psych::omega(CLS_bECRav, nfactors=1, seed=1)

which( colnames(CLS)=="bB5P01")

which( colnames(CLS)=="bB5Pr06")

which( colnames(CLS)=="bB5P11")

which( colnames(CLS)=="bB5P16")

which( colnames(CLS)=="bB5Pr21")

which( colnames(CLS)=="bB5P26")

which( colnames(CLS)=="bB5Pr31")

which( colnames(CLS)=="bB5P36")

CLS_bExtra<- CLS[,c(27,1097,37,42,1098,52,1099,62)]

psych::omega(CLS_bExtra, nfactors=1, seed=1)

#Extra: B5P1,6r,11,16,21r,26,31r,36

which( colnames(CLS)=="bB5Pr02")

which( colnames(CLS)=="bB5P07")

which( colnames(CLS)=="bB5Pr12")

which( colnames(CLS)=="bB5P17")

which( colnames(CLS)=="bB5P22")

which( colnames(CLS)=="bB5Pr27")

which( colnames(CLS)=="bB5P32")

which( colnames(CLS)=="bB5Pr37")

which( colnames(CLS)=="bB5P42")

CLS_bAgree<- CLS[,c(33,43,48,58,68,1100:1103)]

psych::omega(CLS_bAgree, nfactors=1, seed=1)

#Agree: B5P2r,7,12r,17,22,27r,32,37r,42

which( colnames(CLS)=="bB5P03")

which( colnames(CLS)=="bB5Pr08")

which( colnames(CLS)=="bB5P13")

which( colnames(CLS)=="bB5Pr18")

which( colnames(CLS)=="bB5Pr23")

which( colnames(CLS)=="bB5P28")

which( colnames(CLS)=="bB5P33")

which( colnames(CLS)=="bB5P38")

which( colnames(CLS)=="bB5Pr43")

CLS_bConsc<- CLS[,c(29,39,54,59,64,1104:1107)]

psych::omega(CLS_bConsc, nfactors=1, seed=1)

#Consc: 3,8r,13,18r,23r,28,33,38,43r

which( colnames(CLS)=="bB5P04")

which( colnames(CLS)=="bB5Pr09")

which( colnames(CLS)=="bB5P14")

which( colnames(CLS)=="bB5P19")

which( colnames(CLS)=="bB5Pr24")

which( colnames(CLS)=="bB5P29")

which( colnames(CLS)=="bB5Pr34")

which( colnames(CLS)=="bB5P39")

CLS_bNeuro<- CLS[,c(30,40,45,55,65,1108:1110)]

psych::omega(CLS_bNeuro, nfactors=1, seed=1)

#Neuro: 4,9r,14,19,24r,29,34r,39

which( colnames(CLS)=="bB5P05")

which( colnames(CLS)=="bB5P10")

which( colnames(CLS)=="bB5P15")

which( colnames(CLS)=="bB5P20")

which( colnames(CLS)=="bB5P25")

which( colnames(CLS)=="bB5P30")

which( colnames(CLS)=="bB5Pr35")

which( colnames(CLS)=="bB5P40")

which( colnames(CLS)=="bB5Pr41")

which( colnames(CLS)=="bB5P44")

CLS_bOpen<- CLS[,c(31,36,41,46,51,56,66,70,1111:1112)]

psych::omega(CLS_bOpen, nfactors=1, seed=1)

#Open: 5,10,15,20,25,30,35r,40,41r,44

#Wave 1

which( colnames(CLS1)=="PHQ01")

which( colnames(CLS1)=="PHQ08")

CLS1_PHQ<-CLS1[,c(492:499)]

CLS$PHQ_avg <- rowMeans(CLS[,c(492:499)],na.rm=TRUE)

psych::omega(CLS1_PHQ, nfactors=1, seed=1)

which( colnames(CLS1)=="Zung02")

which( colnames(CLS1)=="Zungr04")

which( colnames(CLS1)=="Zungr06")

which( colnames(CLS1)=="Zung08")

which( colnames(CLS1)=="Zung20")

which( colnames(CLS1)=="Zungr22")

which( colnames(CLS1)=="Zung24")

which( colnames(CLS1)=="Zung36")

CLS1_Zung<-CLS1[,c(1425,1443:1444,1428:1434,1445,1436:1442)]

psych::omega(CLS1_Zung, nfactors=1, seed=1)

#Wave 2

which( colnames(CLS2)=="PHQ01")

which( colnames(CLS2)=="PHQ08")

CLS2_PHQ<-CLS2[,c(492:499)]

psych::omega(CLS2_PHQ, nfactors=1, seed=1)

which( colnames(CLS2)=="Zung02")

which( colnames(CLS2)=="Zungr04")

which( colnames(CLS2)=="Zungr06")

which( colnames(CLS2)=="Zung08")

which( colnames(CLS2)=="Zung20")

which( colnames(CLS2)=="Zungr22")

which( colnames(CLS2)=="Zung24")

which( colnames(CLS2)=="Zung36")

CLS2_Zung<-CLS2[,c(1425,1443:1444,1428:1434,1445,1436:1442)]

psych::omega(CLS2_Zung, nfactors=1, seed=1)

#Wave 3

which( colnames(CLS3)=="PHQ01")

which( colnames(CLS3)=="PHQ08")

CLS3_PHQ<-CLS3[,c(492:499)]

psych::omega(CLS3_PHQ, nfactors=1, seed=1)

which( colnames(CLS3)=="Zung02")

which( colnames(CLS3)=="Zungr04")

which( colnames(CLS3)=="Zungr06")

which( colnames(CLS3)=="Zung08")

which( colnames(CLS3)=="Zung20")

which( colnames(CLS3)=="Zungr22")

which( colnames(CLS3)=="Zung24")

which( colnames(CLS3)=="Zung36")

CLS3_Zung<-CLS3[,c(1425,1443:1444,1428:1434,1445,1436:1442)]

psych::omega(CLS3_Zung, nfactors=1, seed=1)

#Wave 4

which( colnames(CLS4)=="PHQ01")

which( colnames(CLS4)=="PHQ08")

CLS4_PHQ<-CLS4[,c(492:499)]

psych::omega(CLS4_PHQ, nfactors=1, seed=1)

which( colnames(CLS4)=="Zung02")

which( colnames(CLS4)=="Zungr04")

which( colnames(CLS4)=="Zungr06")

which( colnames(CLS4)=="Zung08")

which( colnames(CLS4)=="Zung20")

which( colnames(CLS4)=="Zungr22")

which( colnames(CLS4)=="Zung24")

which( colnames(CLS4)=="Zung36")

CLS4_Zung<-CLS4[,c(1425,1443:1444,1428:1434,1445,1436:1442)]

psych::omega(CLS4_Zung, nfactors=1, seed=1)

#Overall

which( colnames(CLS)=="PHQ01")

which( colnames(CLS)=="PHQ08")

CLS_PHQ<-CLS[,c(492:499)]

psych::omega(CLS_PHQ, nfactors=1, seed=1)

which( colnames(CLS)=="Zung02")

which( colnames(CLS)=="Zungr04")

which( colnames(CLS)=="Zungr06")

which( colnames(CLS)=="Zung08")

which( colnames(CLS)=="Zung20")

which( colnames(CLS)=="Zungr22")

which( colnames(CLS)=="Zung24")

which( colnames(CLS)=="Zung36")

CLS_Zung<-CLS[,c(1425,1443:1444,1428:1434,1445,1436:1442)]

CLS$Zung_avg <- rowMeans(CLS[,c(1425,1443:1444,1428:1434,1445,1436:1442)],na.rm=TRUE)

psych::omega(CLS_Zung, nfactors=1, seed=1)

#Pulling new participants only from each of the first 3 waves

CLS1_new<-subset(CLS1,EMPFIRST>0)

CLS2_new<-subset(CLS2,EMPFIRST>0)

CLS3_new<-subset(CLS3,EMPFIRST>0)

#EFA with the psych package

which( colnames(CLS1_new)=="RMRel01")

which( colnames(CLS1_new)=="RMRel19")

RMOnly<- CLS1_new[,434:452]

CLS1_new$RMRel_avg <- rowMeans(CLS1_new[,c(434:452)],na.rm=TRUE)

mean(na.omit(CLS1_new$RMRel_avg))

sd(na.omit(CLS1_new$RMRel_avg))

psych::omega(RMOnly, nfactors=1, seed=1)

library(psych)

#KMO test of sampling adequacy

KMO(RMOnly)

#Bartlett's test of sphericity

RMOnlyNAOMIT<-na.omit(RMOnly)

cortest.bartlett(RMOnlyNAOMIT,n=213,diag=TRUE) #n here is the number of first time participants with no missing data on the RRS

#EFA

efa <- fa(RMOnly,nfactors=5, fm="pa", rotate="none",seed=1)

#Scree-plot

pca<-prcomp(na.omit(RMOnly),scale=TRUE)

varex_pca=pca$sdev^2/sum(pca$sdev^2)

library(ggplot2)

qplot(c(1:19),varex_pca) +

geom_line()+

xlab("Component Number")+

ylab("Proportion of Variance Explained")+

ylim(0,1)

#Reverse coding 17

CLS$RMRel17r<-6-CLS$RMRel17

##Skewness & Kurtosis

which( colnames(CLS)=="RMRel01")

which( colnames(CLS)=="RMRel02")

which( colnames(CLS)=="RMRel03")

which( colnames(CLS)=="RMRel04")

which( colnames(CLS)=="RMRel05")

which( colnames(CLS)=="RMRel06")

which( colnames(CLS)=="RMRel08")

which( colnames(CLS)=="RMRel09")

which( colnames(CLS)=="RMRel10")

which( colnames(CLS)=="RMRel11")

which( colnames(CLS)=="RMRel12")

which( colnames(CLS)=="RMRel13")

which( colnames(CLS)=="RMRel14")

which( colnames(CLS)=="RMRel15")

which( colnames(CLS)=="RMRel16")

which( colnames(CLS)=="RMRel17r")

which( colnames(CLS)=="RMRel19")

#Calculating average RMRel score

CLS$RMRel_avg <- rowMeans(CLS[,c(434:439,441:449,452,1528)],na.rm=TRUE)

which( colnames(CLS)=="RMRel_avg")

RMRel<-CLS[,c(434:439,441:449,1528,452,1529)]

skew(RMRel)

kurtosi(RMRel)

#Single-level CFA

library(lavaan)

model_mod<- '

theta =~ NA*RMRel01 + l2*RMRel02 + l3*RMRel03 + l4*RMRel04 + l5*RMRel05 + l6*RMRel06 +

l8*RMRel08 + l9*RMRel09 + l10*RMRel10 + l11*RMRel11 + l12*RMRel12 + l13*RMRel13 + l14*RMRel14 + l15*RMRel15 +

l16*RMRel16 + l17*RMRel17r + l19*RMRel19

theta ~~ 1*theta

RMRel01 ~~ rw1*RMRel01

RMRel02 ~~ rw2*RMRel02

RMRel03 ~~ rw3*RMRel03

RMRel04 ~~ rw4*RMRel04

RMRel05 ~~ rw5*RMRel05

RMRel06 ~~ rw6*RMRel06

RMRel08 ~~ rw8*RMRel08

RMRel09 ~~ rw9*RMRel09

RMRel10 ~~ rw10*RMRel10

RMRel11 ~~ rw11*RMRel11

RMRel12 ~~ rw12*RMRel12

RMRel13 ~~ rw13*RMRel13

RMRel14 ~~ rw14*RMRel14

RMRel15 ~~ rw15*RMRel15

RMRel16 ~~ rw16*RMRel16

RMRel17r ~~ rw17*RMRel17r

RMRel19 ~~ rw19*RMRel19

RMRel08 ~~ e1*RMRel09

RMRel13 ~~ e2*RMRel15

RMRel10 ~~ e3*RMRel11

RMRel04 ~~ e4*RMRel05'

#CFAs by wave

#Wave 1

CLS1_new$RMRel17r<-6-CLS1_new$RMRel17

fit_1new<-cfa(model_mod,data=CLS1_new)

summary(fit_1new, fit.measures=TRUE,standardized=TRUE)

View(fit_1new)

#Wave 2 New

CLS2_new$RMRel17r<-6-CLS2_new$RMRel17

fit_2new<-cfa(model_mod,data=CLS2_new)

summary(fit_2new, fit.measures=TRUE,standardized=TRUE)

#Wave 2 All

CLS2$RMRel17r<-6-CLS2$RMRel17

fit_CLS2<-cfa(model_mod,data=CLS2)

summary(fit_CLS2,fit.measures=TRUE,standardized=TRUE)

#Wave 3 New

CLS3_new$RMRel17r<-6-CLS3_new$RMRel17

fit_3new<-cfa(model_mod,data=CLS3_new)

summary(fit_3new, fit.measures=TRUE,standardized=TRUE)

#Wave 3 All

CLS3$RMRel17r<-6-CLS3$RMRel17

fit_CLS3<-cfa(model_mod,data=CLS3)

summary(fit_CLS3,fit.measures=TRUE,standardized=TRUE)

#Wave 4

CLS4$RMRel17r<-6-CLS4$RMRel17

fit_CLS4<-cfa(model_mod,data=CLS4)

summary(fit_CLS4,fit.measures=TRUE,standardized=TRUE)

#Single-level alphas and omegas

#Wave 1

which( colnames(CLS1_new)=="RMRel01")

which( colnames(CLS1_new)=="RMRel06")

which( colnames(CLS1_new)=="RMRel08")

which( colnames(CLS1_new)=="RMRel16")

which( colnames(CLS1_new)=="RMRel19")

which( colnames(CLS1_new)=="RMRel17r")

CLS1_new_RMRel<- CLS1_new[,c(434:439,441:449,452,1524)]

psych::omega(CLS1_new_RMRel, nfactors=1, seed=1)

#Wave 2 New

which( colnames(CLS2_new)=="RMRel01")

which( colnames(CLS2_new)=="RMRel06")

which( colnames(CLS2_new)=="RMRel08")

which( colnames(CLS2_new)=="RMRel16")

which( colnames(CLS2_new)=="RMRel19")

which( colnames(CLS2_new)=="RMRel17r")

CLS2_new_RMRel<- CLS2_new[,c(434:439,441:449,452,1523)]

psych::omega(CLS2_new_RMRel, nfactors=1, seed=1)

#Wave 2 All

which( colnames(CLS2)=="RMRel01")

which( colnames(CLS2)=="RMRel06")

which( colnames(CLS2)=="RMRel08")

which( colnames(CLS2)=="RMRel16")

which( colnames(CLS2)=="RMRel19")

which( colnames(CLS2)=="RMRel17r")

CLS2_RMRel<- CLS2[,c(434:439,441:449,452,1523)]

psych::omega(CLS2_RMRel, nfactors=1, seed=1)

#Wave 3 New

which( colnames(CLS3_new)=="RMRel01")

which( colnames(CLS3_new)=="RMRel06")

which( colnames(CLS3_new)=="RMRel08")

which( colnames(CLS3_new)=="RMRel16")

which( colnames(CLS3_new)=="RMRel19")

which( colnames(CLS3_new)=="RMRel17r")

CLS3_new_RMRel<- CLS3_new[,c(434:439,441:449,452,1523)]

psych::omega(CLS3_new_RMRel, nfactors=1, seed=1)

#Wave 3 All

which( colnames(CLS3)=="RMRel01")

which( colnames(CLS3)=="RMRel06")

which( colnames(CLS3)=="RMRel08")

which( colnames(CLS3)=="RMRel16")

which( colnames(CLS3)=="RMRel19")

which( colnames(CLS3)=="RMRel17r")

CLS3_RMRel<- CLS3[,c(434:439,441:449,452,1523)]

psych::omega(CLS3_RMRel, nfactors=1, seed=1)

#Wave 4

which( colnames(CLS4)=="RMRel01")

which( colnames(CLS4)=="RMRel06")

which( colnames(CLS4)=="RMRel08")

which( colnames(CLS4)=="RMRel16")

which( colnames(CLS4)=="RMRel19")

which( colnames(CLS4)=="RMRel17r")

CLS4_RMRel<- CLS4[,c(434:439,441:449,452,1523)]

psych::omega(CLS4_RMRel, nfactors=1, seed=1)

#Multilevel Confirmatory Factor Analysis

mcfa.mod = '

# loadings

level: 1

theta =~ l1*RMRel01 + l2*RMRel02 + l3*RMRel03 + l4*RMRel04 + l5*RMRel05 + l6*RMRel06 +

l8*RMRel08 + l9*RMRel09 + l10*RMRel10 + l11*RMRel11 + l12*RMRel12 + l13*RMRel13 + l14*RMRel14 + l15*RMRel15 +

l16*RMRel16 + l17*RMRel17r + l19*RMRel19

theta ~~ 1*theta

RMRel01 ~~ rw1*RMRel01

RMRel02 ~~ rw2*RMRel02

RMRel03 ~~ rw3*RMRel03

RMRel04 ~~ rw4*RMRel04

RMRel05 ~~ rw5*RMRel05

RMRel06 ~~ rw6*RMRel06

RMRel08 ~~ rw8*RMRel08

RMRel09 ~~ rw9*RMRel09

RMRel10 ~~ rw10*RMRel10

RMRel11 ~~ rw11*RMRel11

RMRel12 ~~ rw12*RMRel12

RMRel13 ~~ rw13*RMRel13

RMRel14 ~~ rw14*RMRel14

RMRel15 ~~ rw15*RMRel15

RMRel16 ~~ rw16*RMRel16

RMRel17r ~~ rw17*RMRel17r

RMRel19 ~~ rw19*RMRel19

RMRel08 ~~ we1*RMRel09

RMRel10 ~~ we2*RMRel11

RMRel13 ~~ we3*RMRel15

RMRel04 ~~ we4*RMRel05

#RMRel02 ~~ we5*RMRel05 #added second time around

level: 2

beta =~ b1*RMRel01 + b2*RMRel02 + b3*RMRel03 + b4*RMRel04 + b5*RMRel05 + b6*RMRel06 +

b8*RMRel08 + b9*RMRel09 + b10*RMRel10 + b11*RMRel11 + b12*RMRel12 + b13*RMRel13 + b14*RMRel14 + b15*RMRel15 +

b16*RMRel16 + b17*RMRel17r + b19*RMRel19

beta ~~ 1*beta

RMRel01 ~~ rb1*RMRel01

RMRel02 ~~ rb2*RMRel02

RMRel03 ~~ rb3*RMRel03

RMRel04 ~~ rb4*RMRel04

RMRel05 ~~ rb5*RMRel05

RMRel06 ~~ rb6*RMRel06

RMRel08 ~~ rb8*RMRel08

RMRel09 ~~ rb9*RMRel09

RMRel10 ~~ rb10*RMRel10

RMRel11 ~~ rb11*RMRel11

RMRel12 ~~ rb12*RMRel12

RMRel13 ~~ rb13*RMRel13

RMRel14 ~~ rb14*RMRel14

RMRel15 ~~ rb15*RMRel15

RMRel16 ~~ rb16*RMRel16

RMRel17r ~~ rb17*RMRel17r

RMRel19 ~~ rb19*RMRel19

RMRel08 ~~ be1*RMRel09

RMRel13 ~~ be2*RMRel15

RMRel10 ~~ be3*RMRel11

RMRel04 ~~ be4*RMRel05

#RMRel02 ~~ be5*RMRel05 #added second time around

# within reliability

numw := (l1+l2+l3+l4+l5+l6+l8+l9+l10+l11+l12+l13+l14+l15+l16+l17+l19)^2

denomw := ((l1+l2+l3+l4+l5+l6+l8+l9+l10+l11+l12+l13+l14+l15+l16+l17+l19)^2)

+(rw1+rw2+rw3+rw4+rw5+rw6+rw8+rw9+rw10+rw11+rw12+rw13+rw14+rw15+rw16+rw17+rw19)

omegaw := numw/denomw

# between reliability

numb := (b1+b2+b3+b4+b5+b6+b8+b9+b10+b11+b12+b13+b14+b15+b16+b17+b19)^2

denomb := ((b1+b2+b3+b4+b5+b6+b8+b9+b10+b11+b12+b13+b14+b15+b16+b17+b19)^2)

+(rb1+rb2+rb3+rb4+rb5+rb6+rb8+rb9+rb10+rb11+rb12+rb13+rb14+rb15+rb16+rb17+rb19)

omegab := numb/denomb

'

mcfa.mod2 <- sem(mcfa.mod, data=CLS, std.lv=T, cluster="id2", estimator="MLR")

summary(mcfa.mod2,fit.measures=TRUE)

#SPL: Construct a null MCFA model to get estimates to total variance

mcfa.null = '

# loadings

level: 1

theta =~ 0*RMRel01 + 0*RMRel02 + 0*RMRel03 + 0*RMRel04 + 0*RMRel05 + 0*RMRel06 +

0*RMRel08 + 0*RMRel09 + 0*RMRel10 + 0*RMRel11 + 0*RMRel12 + 0*RMRel13 + 0*RMRel14 + 0*RMRel15 +

0*RMRel16 + 0*RMRel17r + 0*RMRel19

theta ~~ 1*theta

RMRel01 ~~ rw1*RMRel01

RMRel02 ~~ rw2*RMRel02

RMRel03 ~~ rw3*RMRel03

RMRel04 ~~ rw4*RMRel04

RMRel05 ~~ rw5*RMRel05

RMRel06 ~~ rw6*RMRel06

RMRel08 ~~ rw8*RMRel08

RMRel09 ~~ rw9*RMRel09

RMRel10 ~~ rw10*RMRel10

RMRel11 ~~ rw11*RMRel11

RMRel12 ~~ rw12*RMRel12

RMRel13 ~~ rw13*RMRel13

RMRel14 ~~ rw14*RMRel14

RMRel15 ~~ rw15*RMRel15

RMRel16 ~~ rw16*RMRel16

RMRel17r ~~ rw17*RMRel17r

RMRel19 ~~ rw19*RMRel19

level: 2

beta =~ 0*RMRel01 + 0*RMRel02 + 0*RMRel03 + 0*RMRel04 + 0*RMRel05 + 0*RMRel06 +

0*RMRel08 + 0*RMRel09 + 0*RMRel10 + 0*RMRel11 + 0*RMRel12 + 0*RMRel13 + 0*RMRel14 + 0*RMRel15 +

0*RMRel16 + 0*RMRel17r + 0*RMRel19

beta ~~ 1*beta

RMRel01 ~~ rb1*RMRel01

RMRel02 ~~ rb2*RMRel02

RMRel03 ~~ rb3*RMRel03

RMRel04 ~~ rb4*RMRel04

RMRel05 ~~ rb5*RMRel05

RMRel06 ~~ rb6*RMRel06

RMRel08 ~~ rb8*RMRel08

RMRel09 ~~ rb9*RMRel09

RMRel10 ~~ rb10*RMRel10

RMRel11 ~~ rb11*RMRel11

RMRel12 ~~ rb12*RMRel12

RMRel13 ~~ rb13*RMRel13

RMRel14 ~~ rb14*RMRel14

RMRel15 ~~ rb15*RMRel15

RMRel16 ~~ rb16*RMRel16

RMRel17r ~~ rb17*RMRel17r

RMRel19 ~~ rb19*RMRel19

# within variance

wvar := (rw1+rw2+rw3+rw4+rw5+rw6+rw8+rw9+rw10+rw11+rw12+rw13+rw14+rw15+rw16+rw17+rw19)

# between variance

bvar := (rb1+rb2+rb3+rb4+rb5+rb6+rb8+rb9+rb10+rb11+rb12+rb13+rb14+rb15+rb16+rb17+rb19)

totvar := bvar+wvar

'

mcfa.null2 <- sem(mcfa.null, data=CLS, std.lv=T, cluster="id2", estimator="MLR")

summary(mcfa.null2,fit.measures=TRUE)

#total variance within: 11.615

#total variance between: 21.097

#total overall variance: 32.712

#from actual MCFA:

#remaining error within:

94.751-87.087 # = 7.664

#remaining error between:

230.929-225.207 # = 5.722

#MCFA Variance Accounted for Within-Persons

1-(7.664/11.615) #34%

#MCFA Variance Accounted for Within-Persons

1-(5.722/21.097) #73%

#Convergent and divergent validity with other measures

which( colnames(CLS)=="RMRel01")

which( colnames(CLS)=="RMRel06")

which( colnames(CLS)=="RMRel08")

which( colnames(CLS)=="RMRel16")

which( colnames(CLS)=="RMRel19")

which( colnames(CLS)=="RMRel17r")

RMRel<-CLS[,c(434:439,441:449,452,1528)]

#Means, SDs, and skewness of all measures

mean(na.omit(CLS$RMRel_avg))

sd(na.omit(CLS$RMRel_avg))

skew(CLS$RMRel_avg)

kurtosi(CLS$RMRel_avg)

mean(na.omit(CLS$Timeknwn))

sd(na.omit(CLS$Timeknwn))

skew(CLS$Timeknwn)

kurtosi(CLS$Timeknwn)

#Transforming Timeknwn for non-normality

CLS$Timeknwn_sqrt<-sqrt(CLS$Timeknwn)

mean(na.omit(CLS$Timeknwn_sqrt))

sd(na.omit(CLS$Timeknwn_sqrt))

skew(CLS$Timeknwn_sqrt)

kurtosi(CLS$Timeknwn_sqrt)

mean(na.omit(CLS$bRSE))

sd(na.omit(CLS$bRSE))

skew(CLS$bRSE)

kurtosi(CLS$bRSE)

mean(na.omit(CLS$PHQ_avg))

sd(na.omit(CLS$PHQ_avg))

skew(CLS$PHQ_avg)

kurtosi(CLS$PHQ_avg)

mean(na.omit(CLS$Zung_avg))

sd(na.omit(CLS$Zung_avg))

skew(CLS$Zung_avg)

kurtosi(CLS$Zung_avg)

mean(na.omit(CLS$bAnx))

sd(na.omit(CLS$bAnx))

skew(CLS$bAnx)

kurtosi(CLS$bAnx)

mean(na.omit(CLS$bAvoid))

sd(na.omit(CLS$bAvoid))

skew(CLS$bAvoid)

kurtosi(CLS$bAvoid)

mean(na.omit(CLS$bExtrav))

sd(na.omit(CLS$bExtrav))

skew(CLS$bExtrav)

kurtosi(CLS$bExtrav)

mean(na.omit(CLS$bAgree))

sd(na.omit(CLS$bAgree))

skew(CLS$bAgree)

kurtosi(CLS$bAgree)

mean(na.omit(CLS$bConsc))

sd(na.omit(CLS$bConsc))

skew(CLS$bConsc)

kurtosi(CLS$bConsc)

mean(na.omit(CLS$bNeuro))

sd(na.omit(CLS$bNeuro))

skew(CLS$bNeuro)

kurtosi(CLS$bNeuro)

mean(na.omit(CLS$bOpen))

sd(na.omit(CLS$bOpen))

skew(CLS$bOpen)

kurtosi(CLS$bOpen)

#cor.test(CLS$RMRel_avg,CLS$Timeknwn)

cor.test(CLS$RMRel_avg,CLS$Timeknwn_sqrt)

cor.test(CLS$RMRel_avg,CLS$bRSE)

cor.test(CLS$RMRel_avg,CLS$PHQ_avg)

cor.test(CLS$RMRel_avg,CLS$Zung_avg)

cor.test(CLS$RMRel_avg,CLS$bAnx)

cor.test(CLS$RMRel_avg,CLS$bAvoid)

cor.test(CLS$RMRel_avg,CLS$bExtrav)

cor.test(CLS$RMRel_avg,CLS$bAgree)

cor.test(CLS$RMRel_avg,CLS$bConsc)

cor.test(CLS$RMRel_avg,CLS$bNeuro)

cor.test(CLS$RMRel_avg,CLS$bOpen)

#cor.test(CLS$Timeknwn,CLS$bRSE)

#cor.test(CLS$Timeknwn,CLS$PHQ_avg)

#cor.test(CLS$Timeknwn,CLS$Zung_avg)

#cor.test(CLS$Timeknwn,CLS$bAnx)

#cor.test(CLS$Timeknwn,CLS$bAvoid)

#cor.test(CLS$Timeknwn,CLS$bExtrav)

#cor.test(CLS$Timeknwn,CLS$bAgree)

#cor.test(CLS$Timeknwn,CLS$bConsc)

#cor.test(CLS$Timeknwn,CLS$bNeuro)

#cor.test(CLS$Timeknwn,CLS$bOpen)

cor.test(CLS$Timeknwn_sqrt,CLS$bRSE)

cor.test(CLS$Timeknwn_sqrt,CLS$PHQ_avg)

cor.test(CLS$Timeknwn_sqrt,CLS$Zung_avg)

cor.test(CLS$Timeknwn_sqrt,CLS$bAnx)

cor.test(CLS$Timeknwn_sqrt,CLS$bAvoid)

cor.test(CLS$Timeknwn_sqrt,CLS$bExtrav)

cor.test(CLS$Timeknwn_sqrt,CLS$bAgree)

cor.test(CLS$Timeknwn_sqrt,CLS$bConsc)

cor.test(CLS$Timeknwn_sqrt,CLS$bNeuro)

cor.test(CLS$Timeknwn_sqrt,CLS$bOpen)

cor.test(CLS$bRSE,CLS$PHQ_avg)

cor.test(CLS$bRSE,CLS$Zung_avg)

cor.test(CLS$bRSE,CLS$bAnx)

cor.test(CLS$bRSE,CLS$bAvoid)

cor.test(CLS$bRSE,CLS$bExtrav)

cor.test(CLS$bRSE,CLS$bAgree)

cor.test(CLS$bRSE,CLS$bConsc)

cor.test(CLS$bRSE,CLS$bNeuro)

cor.test(CLS$bRSE,CLS$bOpen)

cor.test(CLS$PHQ_avg,CLS$Zung_avg)

cor.test(CLS$PHQ_avg,CLS$bAnx)

cor.test(CLS$PHQ_avg,CLS$bAvoid)

cor.test(CLS$PHQ_avg,CLS$bExtrav)

cor.test(CLS$PHQ_avg,CLS$bAgree)

cor.test(CLS$PHQ_avg,CLS$bConsc)

cor.test(CLS$PHQ_avg,CLS$bNeuro)

cor.test(CLS$PHQ_avg,CLS$bOpen)

cor.test(CLS$Zung_avg,CLS$bAnx)

cor.test(CLS$Zung_avg,CLS$bAvoid)

cor.test(CLS$Zung_avg,CLS$bExtrav)

cor.test(CLS$Zung_avg,CLS$bAgree)

cor.test(CLS$Zung_avg,CLS$bConsc)

cor.test(CLS$Zung_avg,CLS$bNeuro)

cor.test(CLS$Zung_avg,CLS$bOpen)

cor.test(CLS$bAnx,CLS$bAvoid)

cor.test(CLS$bAnx,CLS$bExtrav)

cor.test(CLS$bAnx,CLS$bAgree)

cor.test(CLS$bAnx,CLS$bConsc)

cor.test(CLS$bAnx,CLS$bNeuro)

cor.test(CLS$bAnx,CLS$bOpen)

cor.test(CLS$bAvoid,CLS$bExtrav)

cor.test(CLS$bAvoid,CLS$bAgree)

cor.test(CLS$bAvoid,CLS$bConsc)

cor.test(CLS$bAvoid,CLS$bNeuro)

cor.test(CLS$bAvoid,CLS$bOpen)

cor.test(CLS$bExtrav,CLS$bAgree)

cor.test(CLS$bExtrav,CLS$bConsc)

cor.test(CLS$bExtrav,CLS$bNeuro)

cor.test(CLS$bExtrav,CLS$bOpen)

cor.test(CLS$bAgree,CLS$bConsc)

cor.test(CLS$bAgree,CLS$bNeuro)

cor.test(CLS$bAgree,CLS$bOpen)

cor.test(CLS$bConsc,CLS$bNeuro)

cor.test(CLS$bConsc,CLS$bOpen)

cor.test(CLS$bNeuro,CLS$bOpen)

#Roommate Relationship Scale Trajectories

library(lme4)

library(lmerTest)

CLS$WAVE.f <-as.factor(CLS$WAVE)

is.factor(CLS$WAVE.f)

#By wave

RMRel_catE <- lmerTest::lmer(RMRel_avg~WAVE.f+EMPFIRST+(1|dyadnum)+(1|id2),data=CLS)

summary(RMRel_catE)

#ICC ID

0.1718/(0.1718+0.7342+0.2350)

#ID Variance

0.1718

#ICC DYAD

0.7342/(0.1718+0.7342+0.2350)

#DYAD Variance

0.7342

#Testing random effects for significance

#Remove ID random intercept

RMRel_catE_noid <- lmerTest::lmer(RMRel_avg~WAVE.f+EMPFIRST+(1|dyadnum),data=CLS)

#Remove ID random intercept

RMRel_catE_nodyad <- lmerTest::lmer(RMRel_avg~WAVE.f+EMPFIRST+(1|id2),data=CLS)

#Compare both to full model

anova(RMRel_catE,RMRel_catE_noid)

anova(RMRel_catE,RMRel_catE_nodyad)

#Semester and seasonal differences

CLS$fall <- cut(CLS$WAVE, br=c(0,2.5,5), labels=c(1,0))

CLS$early <- cut(CLS$WAVE, br=c(0,1.5,2.5,3.5,5), labels=c(1,0,1,0))

is.factor(CLS$fall)

is.factor(CLS$early)

RMRel_earlyfallE <- lmerTest::lmer(RMRel_avg~fall+early+EMPFIRST+(1|dyadnum)+(1|id2),data=CLS)

summary(RMRel_earlyfallE)

#ICC ID

0.1720/(0.1720+0.7347+0.2348)

#ID Variance

0.1720

#ICC DYAD

0.7347/(0.1720+0.7347+0.2348)

#DYAD Variance

0.7347

#Testing random effects for significance

#Remove ID random intercept

RMRel_earlyfallE_noid <- lmerTest::lmer(RMRel_avg~fall+early+EMPFIRST+(1|dyadnum),data=CLS)

#Remove ID random intercept

RMRel_earlyfallE_nodyad <- lmerTest::lmer(RMRel_avg~fall+early+EMPFIRST+(1|id2),data=CLS)

#Compare both to full model

anova(RMRel_earlyfallE,RMRel_earlyfallE_noid)

anova(RMRel_earlyfallE,RMRel_earlyfallE_nodyad)

#Estimating linear effect model

CLS$WAVE0 <- CLS$WAVE-1

RMRel_linearE <- lmerTest::lmer(RMRel_avg~WAVE0+EMPFIRST+(1|dyadnum)+(1|id2),data=CLS)

summary(RMRel_linearE)

#ICC ID

0.1710/(0.1710+0.7334+0.2360)

#ID Variance

0.1710

#ICC DYAD

0.7334/(0.1710+0.7334+0.2360)

#DYAD Variance

0.7334

#Testing random effects for significance

#Remove ID random intercept

RMRel_linearE_noid <- lmer(RMRel_avg~WAVE0+EMPFIRST+(1|dyadnum),data=CLS)

#Remove ID random intercept

RMRel_linearE_nodyad <- lmer(RMRel_avg~WAVE0+EMPFIRST+(1|id2),data=CLS)

#Compare both to full model

anova(RMRel_linearE,RMRel_linearE_noid)

anova(RMRel_linearE,RMRel_linearE_nodyad)

#Comparing categorical, early/Spring, and linear models

anova(RMRel_earlyfallE,RMRel_catE)

anova(RMRel_earlyfallE,RMRel_linearE)

anova(RMRel_catE,RMRel_linearE)

1. Discrepancy between overall sample size and total frequency of responses reflects participants who did not respond to this question. [↑](#footnote-ref-1)
2. Participants selected one or more degrees achieved by their parent with the most education. [↑](#footnote-ref-2)
